# Variability of the UHF Signals Generated by Partial Discharges in Mineral Oil

**DOI:** 10.3390/s19061392

**Published:** 2019-03-21

**Authors:** Michal Kunicki

**Affiliations:** Opole University of Technology, ul.Proszkowska 76, 45-758 Opole, Poland; m.kunicki@po.opole.pl

**Keywords:** partial discharges, UHF, electrical insulation, dielectrics

## Abstract

The paper presents the results of the analysis on the variability of the ultra-high frequency (UHF) signals generated by partial discharges (PD) under the long-term AC voltage. Surface PD (SD) are generated by model PD source (PDS) immersed in brand new mineral oil. Three scenarios are compared and investigated, where different solid dielectrics are applied: pressboard paper (PBP), polytetrafluoroethylene (PTFE) and glass-ceramic (GLS). The PDS is powered continuously by the AC voltage with its relative level of 1.3 of the inception voltage (*U*_i_) within 168 h. UHF signals generated by the continuously occurred SD within 168 h are registered. Various indicators describing the variability of the UHF signals emitted by SD are assigned and analyzed in order to discover if there are any relevant trends presented. Furthermore, some long-term characteristics of the UHF signals emitted by the applied PDS are also announced. As a result, some relevant trends are discovered and related to the properties of the applied dielectric materials, thus the variability of the UHF signals emitted by SD is confirmed. The highest variability of the UHF signals is associated with PBP and the first 48 h after PD inception. Moreover achieved results may be potentially applied for modeling of the PD variability in time, which may be useful for works that concern the development of the UHF method.

## 1. Introduction

PD may be defined as micro discharges within the dielectrics that occur in the presence of the high tangential electric field stress. One of the most common reasons for the PDs appearance is an aging process of the insulation system that is usually resulted by cracks, voids, contaminations and other imperfections, within insulation materials. Furthermore, PD is also recognized as one of the most destructive and undesirable phenomena, that in long perspective leads to progressive local deterioration of insulation system and may result in a complete breakdown of the insulation. According to the electrical power system every high voltage (HV) electrical device may be potentially affected by PD [1]. A significant share of all serious failures around the electrical power distribution system is partially or completely intertwined by PDs [1,2]. The PD occurrence in a liquid insulation system is accompanied by numerous physical phenomena that are fundamentals of the contemporary PD testing systems, i.e., current pulses [3,4], electromagnetic wave emission in the UHF range [5,6], light emission [7,8,9], heat emission [10], chemical reactions [11,12] and acoustic emission [13,14]. Of all the mentioned above methods, UHF is one of the most promising, especially regarding the noise resistivity and relative ease of application, especially in on-site conditions. Main application areas of the UHF method for PD analysis in electric power apparatus are detection [15,16,17] and localization of the source [18,19]. It also should be mentioned that several works have been done on a sensitivity check process for the UHF method, so far [20,21].

According to the contemporary state-of-art publications, it has been confirmed that PD varies in time. Numerous aspects of the PD variability in time have been published so far, but most of them deal with electrical method only. In Reference [22] Kiiza et al. investigated the long-term activity with the presence of HV pulses occurring temporarily. They used the electrical method and analyzed phase-resolved PD (PRPD) patterns and PD density within 20 h. According to their results, HV impulses added to the AC voltage at an early stage of the PD activity do not cause any significant change in PRPD patterns. As a result, a visual examination of the oil-impregnated paper shows no explicit alteration on its surface. It was also confirmed that high voltage impulses have a larger impact on the behavior of PRPD patterns than only prolonged PD activity. Authors noticed that such parameters as the total PD charge and PD density decrease with PD duration time, thus it may be considered as a sign of severe degradation of oil-impregnated paper. Cui et al. in References [23,24] and Wei et al. in Reference [25] presented similar research on the long-term PD activity influence on the paper aging. They have used a needle to plate electrode configuration and generated the PD within a few hours and the energy, as well as PD density, have been analyzed. Regarding the authors of References [23,24], the authors showed that the depolymerization of cellulose in pressboard is the main reason for the development of partial discharge, and discharges occur in different positions of pressboard in different deterioration stages. Despite PD were analyzed within 120 min only, three relevant activity regions were pointed on the grounds of the PD amplitudes and PD density time runs: initial, develop and stagnation stages. Similar conclusions are presented in Reference [25]. In the 1st initial stage discharge energy is relatively low then, in the development stage it increases, and in the last pre-breakdown stage energy decreases reaching an approximate level from the initial stage. Other aspects related to the long-term PD activity were presented by Florkowska et al. in Reference [26]—experiments on interactions of conductors with polymeric material at the PD presence within a long period were introduced. A PD evolution based on the PRPD patterns was presented. Authors divided the analyzed period into two stages, that represents different behaviors of PD: during the 1st stage a slowdown of PD activity was noticed at the beginning and at the end of that stage (increased PD activity in the middle of the period), while during the 2nd stage rather low intensity of high charge magnitude pulses was observed in both phase ranges but the total number of discharges was increasing at first part of the period and then a drop in the number of discharges was observed—caused by an irreversible alteration of material surface. Iwashita et al. in Reference [27] presented results of the investigation on the PD variability on the model of the oil-paper cable insulation, where apparent charge and PD density have been analyzed within 14 days. According to their results, a relatively constant slight increase of the PD amplitudes was noticed during the analyzed period and negative streamers were dominant. Which is also interesting rapid increases of PD magnitudes and PD counts were observed in the very first period after PD inception—afterwards, those magnitudes returned to the stable level. Other papers where variability of the electrical signals emitted by PD was investigated are References [28,29]. Authors used the electrical method and showed how the signal emitted by PD under long-term voltage vary in time. In addition, the influence of the insulation oil condition on the emitted signals was investigated.

Despite it is commonly admitted that PD varies in time, minor relevant research may be found where UHF signals emitted by PD are analyzed within the long term. Knowing that the PD itself changes over time, it is essential to be answered how (or if) the signals emitted by the PD in the UHF range are affected by the PD variability. Regarding the crucial aspects related to the PD analysis using the UHF method, the author has proposed complete advanced research on the variability of the UHF signals generated by PD in mineral oil in this paper. Such an approach seems to be essential especially in terms of the proper measurement results interpretation as well as its further potential applications for development of the UHF method.

## 2. Fundamentals of PD

A breakdown process of ideal, pure liquid dielectrics is generally different than the breakdown process for gasses or solids (but some modest similarities may be observed regarding the liquids and gasses) [30,31]. It is also characteristic that all of the real liquids (especially in working conditions) are usually contaminated by various foreign substances such as solids, other liquids and dissolved gasses. Those contaminants significantly influence a breakdown process of the liquid. An initial state of a breakdown process is a PD generation. Few theories of the PD generation in liquid dielectrics may be pointed, i.e., solid particles, electro-convection, gaseous inclusion, liquid globules, cavity. Furthermore, regardless of the generation mechanism a PD is always accompanied by various physical phenomena that are tightly related to different forms of energy e.g., current pulses, chemical reactions (hydrogen and other hydrocarbon gases generation), local heat emission, acoustic wave emission, electromagnetic wave emission, light emission, local pressure adjustments and even ionizing radiation. A special case of a PD is an SD. It may be defined as a form of PD activity associated with a solid dielectric surface while the high tangential electric field stress is present. The crucial aspect of the SD is that it occurs in the abutment of three different environments: galvanic (HV electrode), solid dielectric (barrier between HV and grounded electrodes) and dielectric environment (gas or liquid). As a result, a faster discharge propagation along an insulating surface is possible, also a lower field strength is needed to initiate the PD process compared to oil or gas without the adjacent insulating surface [32]. Regarding the common contemporary knowledge, a rise time of PD pulse is usually in the range of approximately a few ns. Thus, it is expected that electromagnetic wave radiation is generated by such short pulses. Some part of the emitted radiation travels within the galvanic parts connected with the PD source (in both directions: to the supply source as well as to the ground), while the other part is emitted into the surrounding environment. The propagation mechanisms of electromagnetic signals in galvanic parts and in oil inside of the limited space are significantly different. Furthermore, different attenuations of the signals are also related to those materials. For example, in case of oil-filled power transformers signals measured in galvanic parts using electrical method (capacitive or inductive sensors), are influenced by the inductivity of every turn of the winding coil and all stray capacities which works as a low pass filter, thus a measured upper-frequency limit is typically limited to approximately tens of MHz [33]. The propagation of the electromagnetic signals inside of the transformer tank is a radiated emission in the entire volume of the transformer, in oil and pressboard. Thus an original electromagnetic wave emitted by the PD may be attenuated and reflected by metallic parts. Finally, the measured signal is distorted (with reference to the original one) and its frequency band is usually from hundreds of MHz to few GHz (UHF range) [16].

The SD is always affected by numerous physical process related to properties of the solid dielectric material, surrounding dielectric (liquid or gas) and environmental conditions. Any modification of the environment or the SD generation conditions radically affects the final measurement results, thus the stochastic nature of any PD is now the highest challenge from the metrological point of view. Numerous current state-of-art papers deal with environmental conditions influence on the PD generation. In Reference [34] the influence of the electric field strength as a function of the electric potential gradient on the PD generation is presented. The influence of dielectric liquid type (mineral, vegetable, synthetic, silicone, etc.) is tested in References [4,12], while the oil condition (contaminations, corrosive sulfur, gases etc.) is investigated in References [29,35]. Analysis of the electrodes materials and their geometry influence on the PD generation are presented in Reference [34]. Other papers deal with the influence of the temperature [36], pressure [37], voltage harmonics [38] or different voltage types (DC or lightning impulse) [31] on the PD generation.

## 3. Research Methodology

As mentioned in Section 1 the main purpose of the presented research is to investigate the variability of the UHF signals generated by PD under the long-term AC voltage. It was to be achieved by assigning selected long-term characteristics and analyzing their variability and dependency on the PD duration as well as on the applied solid dielectric material. Thus the main idea was to investigate what are the results of the continuously occurred PD and how it behaves (changes) in time—in terms of the parameters measured by the UHF method. Three different PD sources (PDS) were applied in the research, each with a different solid dielectric barrier placed between HV and grounded electrodes: pressboard paper (PBP), polytetrafluoroethylene, so-called Teflone (PTFE) and glass-ceramic (GLS). All of those barriers were 8 mm thick and of the size of 15 cm × 15 cm. The high voltage (HV) electrode of the PDS was 1 cm thick plate made with brass with a diameter of 3 cm, while the grounded (GND) electrode has been 1 cm thick steel plate with a diameter of 12 cm. All edges of both electrodes were 1 mm rounded. The PDS was immersed in a brand new mineral insulation oil—basic physical and chemical properties of the oil before and after measurements are presented in Table 1. Measuring tank was filled with approximately 120 L of oil (60 × 50 × 40 cm). Before each measurement series oil was left in the tank for 24 h in order to reach an equilibrium with the ambient (moisture, bubbles, etc.). Then, oil samples were taken and analyzed before and after each scenario—generally, no significant adjustments in the oil properties have been observed. Furthermore, in order to make the results reliable and comparable special attention was paid to the measurement conditions. Constant environmental conditions have been provided during the experiment: air/oil temperature, humidity and pressure—dedicated air conditioning was used to control all those parameters in the test cell (temperature: 20 °C, humidity: 23%), and temperature of top and bottom of the oil was also monitored independently (also were constant: 20 °C). Moreover the pressure inside the measuring tank was monitored during the research and its constant relative level (in relation to the ambient pressure) was provided by the relevant valves on the upper cover of the tank equipped with the desiccant filters—absolute deviation of the pressure within all measurements were 4 hPa, which was an effect of the atmospheric pressure adjustments, which could not be controlled in the applied setup. Each PDS was placed in exactly the same position, including electrodes and solid barrier positions. The UHF sensor was also positioned in the same place and depth. Thus geometry of the setup during all of the measurement series was not changed. The testing cell was shielded so no additional external interferences were expected during the experiment (which was checked and confirmed by relevant background noise analysis).

Each of the applied PDS was powered continuously by the AC voltage with its relative level of 1.3 of the inception voltage (*U*_i_) of the PDS within 168 h. The *U*_i_ was defined as the voltage at which the apparent charge of PD was higher than 100 pC—1 kV/s automatic controlled voltage ramp was applied. The *U*_i_ was assigned as a mean value of the surveys made on three applied scenarios—each scenario was tested 30 times, and then a mean value was assigned. Regarding each material final values of *U*_i_ were: 23.6, 22.7 and 23.3 kV, for GLS, PTFE and PBP respectively. Those results are approximately 23 ± 0.5 kV, so the value 23 kV was chosen as a reference *U*_i_. As a result, the test voltage was set to 30 kV regarding all scenarios (approximately 1.3 *U*_i_). On the grounds of a number of surveys, the proposed 168 h period was the longest period that allowed PD to be generated continuously as well as the PDS not to breakdown—it was possible to make the period longer but voltage level needed to be lower, which resulted in randomly appeared PD extinctions. Afterwards, UHF signals generated by the continuously occurred PDs within 168 h were registered every 12 h. PD signals within 30,000 cycles of 50 Hz supply voltage were captured during every single measurement. All of the scenarios were repeated with three new samples of the solid dielectric materials, and the tank was always filled with new oil for each sample—results presented and discussed in Section 4 were based on mean values assigned over three measurement series for each material sample (30,000 cycles per each registration for each sample). A narrow band method was applied for the UHF measurements with the integration window bandwidth set to 1.5 MHz. Further post measurement research was generally based on the spectrum analysis of the UHF signals, phase-resolved PD (PRPD) patterns analysis and investigation of the selected qualitative and quantitative descriptors. All experiments proceeded under laboratory conditions.

**Figure 1:**
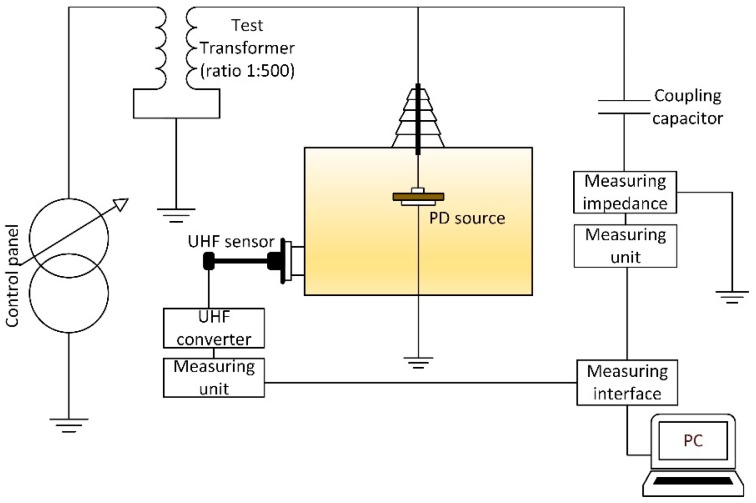
Layout of the measurement setup.

Commercially available MPD600 PD measuring system was used for the research (all of the components are manufactured by Omicron electronics GmbH, Klaus, Austria). The UVS610 sensor, UHF608 converter and MPD600 module were applied (Figure 1). A frequency range of the UHF sensor was from 150 MHz to 1000 MHz and of the UHF608 converter, it was 220 MHz to 850 MHz. The UHF sensor was immersed directly in the oil via measuring valve. Neither the HV level nor the measuring track gain were adjusted during all of the measurements. Additionally, the second measurement track based on the conventional electrical method was used to support the continuous monitoring of the voltage level, apparent charge and voltage phase measurements. Furthermore, a high voltage separation of the measuring equipment was provided by fiber optic connections and battery power supply for all MPD instruments.

## 4. Results and Discussion

This section is divided into three parts and each part investigates different aspects of UHF signals variability within a long-term regarding the three applied PDS. All of the results are commented and discussed around the relevant subsection: results of the spectrum analysis of the UHF signals are presented in Section 4.1, PRPD analysis is yielded in Section 4.2 and selected qualitative and quantitative descriptors are investigated in Section 4.3.

### 4.1. Spectrum Analysis

**Figure 2:**
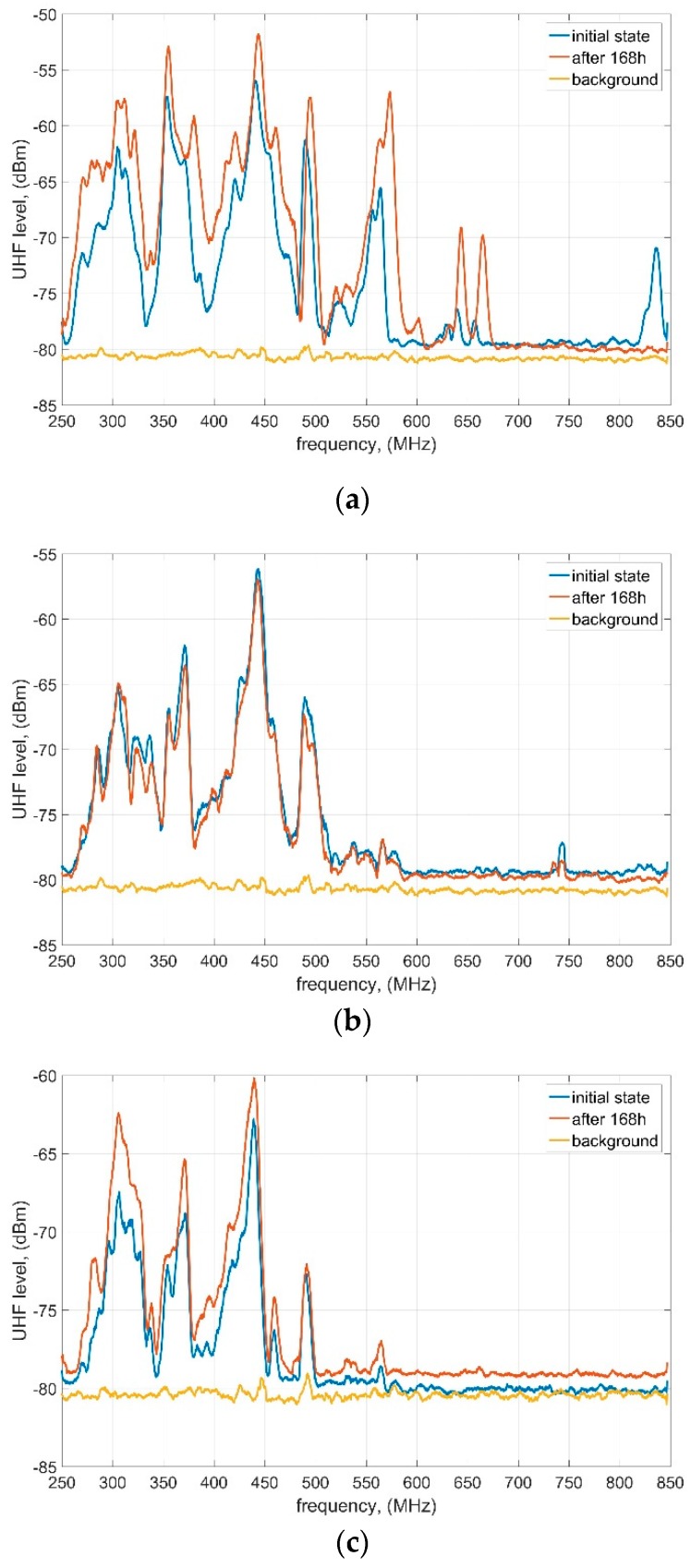
UHF spectrum of the signals emitted by SD at the initial state and after 168 h of continuous PD generation: (**a**) PBP; (**b**) PTFE; (**c**) GLS.

For each of the PDS scenarios, UHF spectrums have been registered at the initial state (5 min after energizing the PDS with relevant voltage level) and at the end of the experiment (after 168 h of continuous PD generation). The comparison of all of the results is presented in Figure 2. The highest activity may be observed around a few center frequencies, no matter what kind of the PDS was applied. Some explicit local peaks around 300 MHz, 380 MHz and 450 MHz may be noticed in all scenarios. In case of the PBP, an additional 550 MHz peak may be observed, while in case of GLS and PTFE a local extremum appears around 500 MHz. It also needs to be emphasized that UHF signals emitted by PBP have wider frequency range (approximately 250–600 MHz) than GLS and PTFE (250–500 MHz). Furthermore, some small activity was noticed around 650 MHz and 830 MHz (initial state only) in PBT scenario. Analyzing the time variability of the characteristics it should be noted that the most stable are signals generated by PTFE, where generally no variability was observed (Figure 2b). Despite some variability may be noticed in the GLS scenario, both of the characteristics are also in accordance with each other—only a few dB rise appears according to the local peaks (Figure 2c). The most significant variation regarding the spectrum of the UHF signals emitted by PD at the beginning and at the end of the experiment is observed in PBP scenario (Figure 2a). Not only some obvious gain of the UHF signals are presented but also an activity is related to wider local frequency bands. Furthermore, an activity band that was presented at the initial state (approximately 830 MHz) disappears at the end of the analyzed period.

As a relevant conclusion of the discussion on the UHF spectrum, total energies of the UHF spectrums detected by the sensor at the initial state and at the end of the experiment (shown in Figure 2) are calculated and presented in Table 2. It is characteristic that in both PBP and GLS scenarios total energy of the spectrum increased during the analyzed period, while in case of PTFE it was reduced for approximately 2 dB. The most significant rise is observed in PBP scenario—almost 4 dB. Further discussion on the energy is provided in Section 4.3.

It is expected that all of the results observed and described above are caused mainly by the different properties of the dielectric materials that were applied as a solid barrier in each PDS. Presented observations are related to the sensibility of the applied materials to molecular degradation caused by the long-term SD (and HV as well) exposure. Of all those three materials, PBP is the most prone to surface treeing which results in the highest variability of the emitted signals. On the other hand, due to its molecular structure, PTFE seems to be the most impervious to degradation of its surface by long term SD—PD easily “slides” across its surface not affecting it significantly, thus the PD generation conditions are relatively constant within the whole tested period (only local oil degradation and electrodes erosion are expected to be potentially the most relevant).

### 4.2. PRPD Patterns Analysis

**Figure 3:**
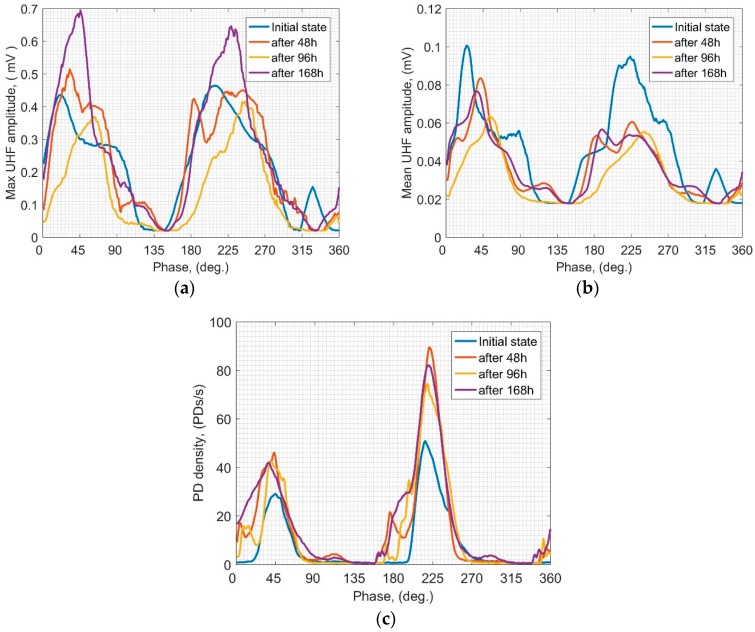
Means over PRPD patterns of the UHF signals emitted by SD for PBP scenario: (**a**) max UHF amplitude; (**b**) mean UHF amplitude; (**c**) PD density.

In this subsection PRPD patterns achieved for all of the applied PDS in relevant time steps are presented and discussed. All of the PRPD were assigned on the grounds of the UHF signals captured for the frequency of 450 MHz (with an integration window width of 1.5 MHz), which was identified as providing the highest signal to noise ratio regarding all PDS. As mentioned in Section 3 an additional measuring track was used for the proper UHF signals synchronization with the phase of the supply voltage. Results of the PRPD for PBP are shown in Figure 3. The zones of highest activity in the phase domain are associated with relatively constant angles: approximately 30–70° and 210–250°, while max values appear around 45° and 225° in case of all analyzed parameters. Regarding the max UHF amplitudes it may be noticed that after first 48 h all of the registered values decrease, and only at the last point (after 168 h) they raised again. In addition, the behaviors in 1st and 2nd half-cycles are similar. In case of the mean amplitude, a rather constant decreasing trend may be noticed in both half-cycles—only according to the 1st half-cycle a slight rise at the last registration point may be observed. After 48 h mean amplitudes in 1st half are significantly higher than those in 2nd, which was not observed in case of max amplitudes. Analyzing the PD density, the only explicit observation is that most of the PD events are related to the 2nd half, where the highest variability is also noticed. It is because the PD generation had been initiated by negative streamer (or anode-directed) mechanism which may be commonly observed in liquids. Negative charges create “memory charge” that affects the subsequent discharge via the field distortion effect. As a result, negative streamers appear much easier (higher number of negative charges) and require lower energy than positive ones to be injected [24,26]. Furthermore, it should be emphasized that based on the PRPD analysis one can point to the rather random behavior of PD density—no explicit trend is identified. These results partially confirm discussion presented in the previous subsection—PBP is quite prone to erosion caused by SD which may be also observed in the variability of the emitted signals in the phase domain.

**Figure 4:**
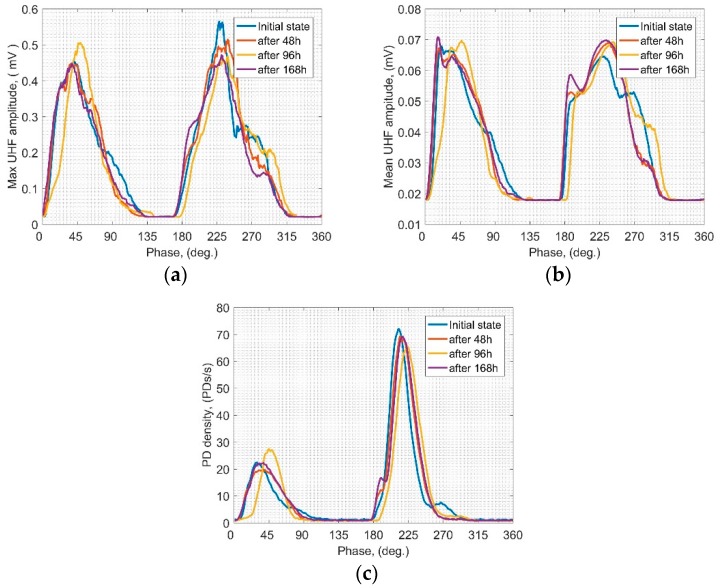
Means over PRPD patterns of the UHF signals emitted by SD for PTFE scenario: (**a**) max UHF amplitude; (**b**) mean UHF amplitude; (**c**) PD density.

PRPD results achieved for the PSD equipped with PTFE are presented in Figure 4. At a glance, one can see that signals registered in the relevant time steps are very similar to each other. Regarding both max and mean UHF amplitudes, their behavior in the 1st and 2nd half is similar in relation to the bandwidth of the phase domain as well as the local peaks. It is only in case of the 2nd half of max amplitude (Figure 4a) where a decreasing trend may be noticed along with the time. Analyzing the PD density (Figure 4c) it also may be pointed out that higher PD activity is associated with 2nd half, and it is almost constant within all of the analyzed periods. Despite some differences between PRPDs obtained for the PTFE scenario in the analyzed time steps, it is generally confirmed that PTFE is relatively more resistant to SD erosion than other solid dielectrics investigated in this study.

**Figure 5:**
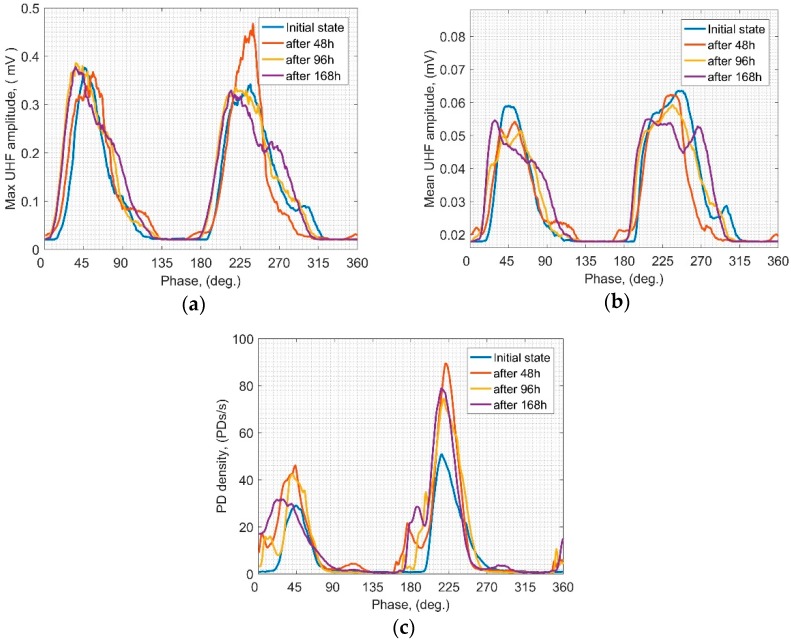
Means over PRPD patterns of the UHF signals emitted by SD for GLS scenario: (**a**) max UHF amplitude; (**b**) mean UHF amplitude; (**c**) PD density.

The last of the analyzed PRPD scenario was GLS. Representative results showing potential variability of the UHF signals generated by SD under a long-term AC voltage in the phase domain are presented in Figure 5. Compared to previous scenarios, a quite moderate variability may be noticed. The highest development is observed regarding the PD density (Figure 5c)—a rapid rise within the first 48 h (peak) and a stabilizing trend afterwards is presented. Similar behavior may also be observed in case of the 2nd half of the max UHF amplitude (Figure 5b), while behavior in the 1st half is rather constant. A characteristic slow decreasing trend is presented in Figure 5b, where mean amplitudes are illustrated.

When comparing all three presented scenarios regarding the analysis of PRPD patterns, it should be emphasized that the PTFE and GLS scenarios have shown a similar range of the potential variability. It is also confirmed that those materials are significantly less prone to the degradation influence of SD, which was also observed in hardly visible variability of the registered UHF signals (however, generally they also are affected by SD). Thereby UHF signals emitted by SD with PBP solid barrier have been found to vary explicitly along with the duration of the SD. Therefore on the current stage of the research, it may be stated that UHF signals generated by SD vary in time (no matter what dielectric is applied) and the range of their variability significantly depends on the material of the solid barrier.

### 4.3. Descriptors Analysis

**Figure 6:**
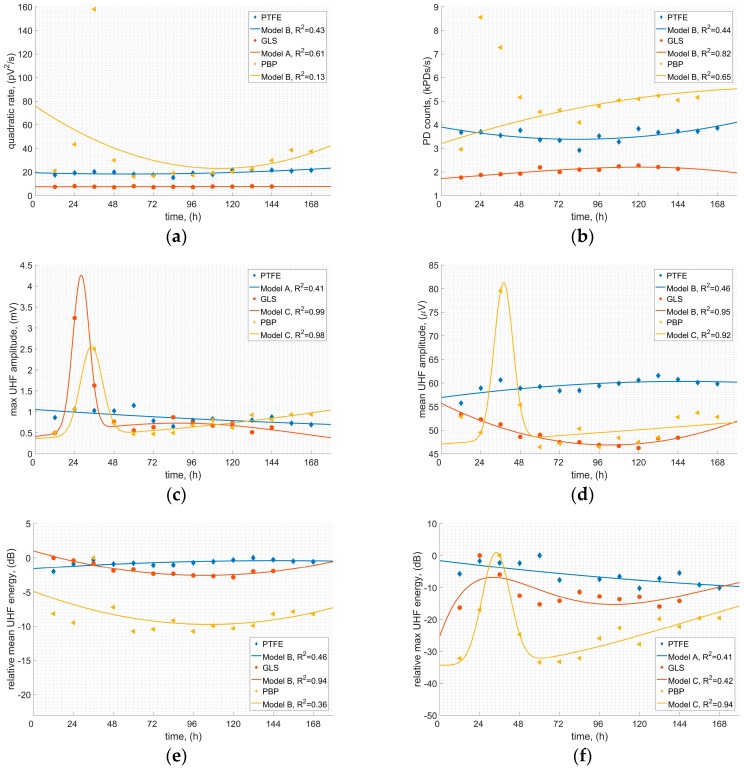
Variability of the selected descriptors of the UHF signals emitted by SD within 168 h: (**a**) quadratic rate; (**b**) PD counts; (**c**) max UHF amplitude; (**d**) mean UHF amplitude; (**e**) relative mean UHF energy; (**f**) relative max UHF energy.

The last analysis was dedicated to surveys on qualitative and quantitative descriptors. Six descriptors were investigated: max UHF amplitude, mean UHF amplitude, PD density (PD counts), quadratic rate, mean and max relative energy (assigned as the mean/max cumulative energy of the UHF pulse in particular time step, related to the maximum value of the energy within the whole analyzed period, and expressed in dB). All those descriptors are commonly applied to assess PD activity in an electrical apparatus, so it is essential to confirm if they show any variability regarding the analysis presented in previous subsections. Results are presented in Figure 6. Each of the plots contains time characteristics of the relevant descriptor according to three SD scenarios. Additionally, some simple regression models were assigned, and they are presented in the plots in order to support the interpretation of the results: to estimate if there are any trends discovered within the achieved characteristics (increasing, decreasing, constant, random). Modeling functions that were applied: A—linear, B—2nd order polynomial, C—2nd order Gaussian model. Relevant modeling functions used in the plots are indicated in the legends.

Variability of the quadratic rate is provided in Figure 6a. Almost constant characteristics may be observed regarding the GLS and PTFE scenario, while an obvious local peak (not included in the model) is noticed in case of the PBP: a rapid gain and drop are noticed within the first 48 h, whereupon a stabilized, a slight rising trend may be seen. Similar behaviors are observed in all of the analyzed descriptors regarding the PBP scenario. In case of the PD counts analysis (Figure 6b) neither PTFE nor GLS shows any significant variability—GLS showed a very slight rising trend until the last two measuring points where it starts to fall; PTFE is characterized by a constant decreasing trend within the first 84 h, and then symmetrical rising slope is noticed (after 168 h PD count is the same as at the beginning of the test). In PBP scenario a visible raising trend is presented after 48 h (after a “peak” region). The most interesting results are presented in Figure 6c, where the max UHF amplitude is investigated. Two of the analyzed scenarios show an evident local peak within the first 48 h—not only the PBP but also GLS. As for PTFE, a slight downward trend is discovered. Long-term characteristics of the mean UHF amplitudes are presented in Figure 6d. Apart from the local peak in the PBP scenario, there is also an explicit downward trend visible in GLS scenario as well as a slight upward trend in PTFE scenario.

Regarding the energy patterns analysis (Figure 6e,f), some local peaks in the PBP scenario are also observed. The highest energy is observed at 36 h after the system was energized. Afterwards, generated energy rapidly drops reaching the initial state level, and after approximately next 24 h relatively constant upward trend is observed. A quite similar situation may be noticed regarding the max energy in GLS scenario, but the trend is not so obvious. In case of the mean relative energy in PTFE scenario almost constant values are observed (±1 dB), while max energy consequently decreases with time (Figure 6f). These observations confirm the previous analysis on the spectrum energy, provided in Section 4.1. It is characteristic that total spectrum energy behaves similar to the max pulse energy regarding all scenarios since observed trends are consistent.

The results presented in that subsection have confirmed most of the observations and conclusions made on the previous analysis. PBP has confirmed to be the most prone for the SD erosion which results in the highest UHF signal variability, especially at the first stage after the PD inception. PTFE scenario showed that despite such PDS emits the most stable UHF signals within a long-term, those signals also vary in time.

## 5. Conclusions

Results presented in this paper have allowed us to formulate several conclusions and significant observations that should be emphasized—among all the major achievements of the presented research are:

- Confirmed that UHF signals generated by SD vary in time no matter what dielectric material is used as a solid barrier and the very first period after PD inception is essential regarding the observed variabilities.
- Confirmed that SD generated on each of the applied solid dielectric materials emits different spectrum in the UHF range—the most significant differences are noticed according to the total energy of the UHF spectrum.
- UHF signals emitted by SD when analyzing in the long-term perspective show different variabilities, that depend on the applied material.
- Confirmed that PBP is the most prone to degradation by SD which results in the highest variability of the emitted UHF signals.
- Regarding the PBP all of the analyzed descriptors showed a significant local peak within the first 48 h.
- Confirmed that UHF signals emitted by prolonged SD on PTFE are the least variable from among all investigated scenarios—although they also vary.
- The interpretation process of the UHF signals may be enhanced if information about the duration of the analyzed PD is available (e.g., extracted from the on-line PD monitoring system if available).

According to the presented research, it may not be explicitly pointed what is the exact expected behavior of the UHF signals within a long period, especially in perspective much longer than the analyzed period. Nevertheless, some of the previously presented results, dealing with electrical method and published within other papers are confirmed: the highest variability in signals generated by PD is associated with every first period after the inception, afterwards a relatively stable behavior is generally noticed. The analyzed UHF signals have shown similar behavior to the electric signals discussed by other papers mentioned in Section 1. Thus further research is needed prior to relevant modeling of the PD behavior regarding not only the UHF method but also other commonly applied methods. However, it has been pointed that UHF signals emitted by applied SD vary in time and mainly in case of the PBP the first 48 h after the inception of the PD is crucial since the variability of the signals is the most significant in that period. Thus it is desired that the beginning of the SD on PBP should be recorded for proper assessment of the PD.

## Figures and Tables

**Table 1:** Basic physical and chemical properties of the applied oil.

Parameter	Value	
	Before Tests	After Tests
Breakdown voltage, (kV)—2.5 mm gap (IEC60156)	64 (relative deviation 19%)	61 (relative deviation 16%)
Dissipation factor—50 °C, (tanδ)	0.0003	0.0005
Water content—Karl-Fischer, (ppm)	29	30
Resistivity 50 °C, (Ωm)	6.2 × 10^11^	5.9 × 10^11^
Neutralization number, (mgKOH/g)	<0.01	<0.01
Flash point, (°C)	141	141

**Table 2:** Total energy of the UHF spectrum emitted by PD and detected by the UHF sensor.

Applied Solid Dielectric	Initial	End
PBP	−35.59 dBm	−31.07 dBm
PTFE	−37.19 dBm	−39.30 dBm
GLS	−42.30 dBm	−39.45 dBm
